# Intrinsic cardiovascular autonomic regulatory system of astronauts exposed long-term to microgravity in space: observational study

**DOI:** 10.1038/npjmgrav.2015.18

**Published:** 2015-11-30

**Authors:** Kuniaki Otsuka, Germaine Cornelissen, Yutaka Kubo, Mitsutoshi Hayashi, Naomune Yamamoto, Koichi Shibata, Tatsuya Aiba, Satoshi Furukawa, Hiroshi Ohshima, Chiaki Mukai

**Affiliations:** 1 Department of Chronomics and Gerontology, Tokyo Women’s Medical University, Tokyo, Japan; 2 Halberg Chronobiology Center, Department of Integrative Biology and Physiology, University of Minnesota, Minneapolis, MN, USA; 3 Department of Medicine, Tokyo Women’s Medical University, Medical Center East, Tokyo, Japan; 4 Space Biomedical Research Group, Japan Aerospace Exploration Agency, Tokyo, Japan

## Abstract

The fractal scaling of the long-term heart rate variability (HRV) reflects the ‘intrinsic’ autonomic regulatory system. Herein, we examine how microgravity on the ISS affected the power-law scaling β (beta) of astronauts during a long-duration (about 6 months) spaceflight. Ambulatory electrocardiographic (ECG) monitoring was performed on seven healthy astronauts (5 men, 52.0±4.2 years of age) five times: before launch, 24±5 (F01) and 73±5 (F02) days after launch, 15±5 days before return (F03), and after return to Earth. The power-law scaling β was calculated as the slope of the regression line of the power density of the MEM spectrum versus frequency plotted on a log_10_–log_10_ scale in the range of 0.0001–0.01 Hz (corresponding to periods of 2.8 h to 1.6 min). β was less negative in space (−0.949±0.061) than on Earth (−1.163±0.075; *P*<0.025). The difference was more pronounced during the awake than during the rest/sleep span. The circadian amplitude and acrophase (phase of maximum) of β did not differ in space as compared with Earth. An effect of microgravity was detected within 1 month (F01) in space and continued throughout the spaceflight. The intrinsic autonomic regulatory system that protects life under serious environmental conditions on Earth is altered in the microgravity environment, with no change over the 6-month spaceflight. It is thus important to find a way to improve conditions in space and/or in terms of human physiology, not to compromise the intrinsic autonomic regulatory system now that plans are being made to inhabit another planet in the near future.

## Introduction

The absence of gravitational stimuli during spaceflight induces a number of adaptive changes within the cardiovascular system that may affect crew health and safety.^1,2^ Most imperative, cardiovascular modifications occurring in microgravity consist of altered blood volume distribution,^3,4^ impaired myocardial properties,^5–7^ and/or vascular remodeling.^8–10^ In addition, the baroreflex in space is chronically unchallenged owing to removal of intravascular hydrostatic pressure gradients.^10–13^ The interplay between baroreflex and hemodynamic and body fluid alterations is likely to affect neural mechanisms involved in dynamic cardiovascular regulation, but the way in which this occurs in astronauts in space is still poorly understood.

During long-duration spaceflight, Norsk *et al.*^14^ reported reductions in blood pressure and in systemic vascular resistance together with increases in cardiac output and stroke volume, but no apparent change in sympathetic nerve activity estimated from venous blood plasma concentration of norepinephrine. Christensen *et al.*^15,16^ noted an increase in platelet norepinephrine and epinephrine in 4 out of 5 cosmonauts studied shortly after exposure to microgravity, contrary to a marked decrease associated with head-down bedrest. The authors interpret their result as a likely increase in sympathoadrenal activity during microgravity, noting that the reason why sympathoadrenal activity does not decrease in space remains to be elucidated.

Human spaceflight dramatically alters cardiovascular demands associated with work loads.^17^ Without appropriate adaptation, marked cardiovascular deconditioning may occur that can be detected by changes in heart rate variability (HRV).^18^ The fractal scaling behavior, including the long-term *β* (beta) or *α* (alpha) HRV, is thought to reflect the ‘intrinsic’ autonomic regulatory system.^19–22^ Early data from the Russian Mir space station suggest that the overall cardiovascular system adapted well to the microgravity environment, with minimal changes in heart rate and blood pressure,^23,24^ or in the scaling exponent *α* during long-term orbital flight. Heartbeat records covering 6 h in space were consistent with those found for healthy subjects on Earth.^25^ To date, however, there have been no investigations of the fractal scaling response to microgravity on the International Space Station (ISS), especially during long-duration spaceflights, our purpose herein.

Specifically, we examine the time course of changes in the power-law scaling *β* of HRV before, during, and after long-duration (about 6 months) spaceflight, during which astronauts were exposed to the microgravity environment of the ISS. Focus was placed on four specific frequency bands of long-term (up to 24-hour) HRV: the high-frequency (HF: >0.15 Hz), low frequency (LF: 0.04–0.15 Hz), very low frequency (VLF: 0.0033–0.04 Hz) and ultralow frequency (ULF: <0.0033 Hz) components.

## Results

### Circadian rhythm in fractal nature of heart rate variability

The recent discovery that a circadian pacemaker has a crucial role in generating fractal patterns in HRV sheds an entirely new light on both fractal control networks and the function of the circadian clock, allowing a bridge to be built between the fields of circadian biology and fractal physiology.^26–28^ Herein, we show that the fractal regulation of HRV follows a circadian rhythm, some of its characteristics affected by microgravity in space.

**Figure 1:**
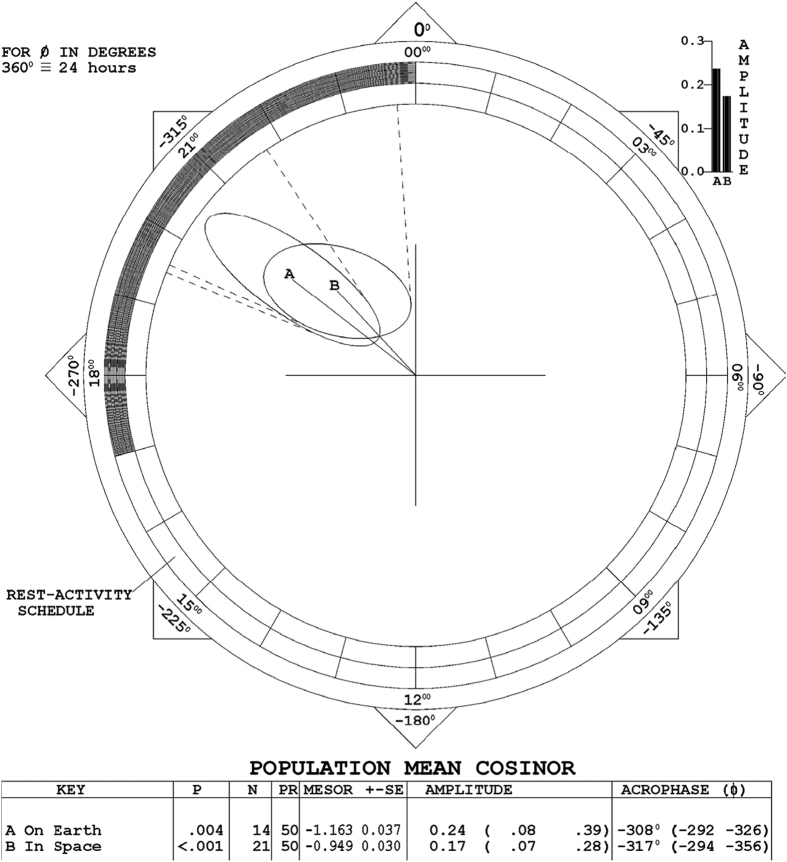
Population-mean cosinor summary of the slope of the power-law scaling of NN intervals, beta, on Earth and in space. Values in parentheses for amplitude and acrophase are 95% confidence limits. MESOR, Midline-Estimating Statistic of Rhythm.

Figure 1 illustrates the average circadian amplitude-acrophase (*A*, *ϕ*) vectors (directed lines originating from the center of the plot) of the seven astronauts monitored on Earth (2 profiles) and in space (three sessions), summarized by population-mean cosinor. The 24-h period is represented as a circle with 360° equated to 24 h, 00:00 hours (top of the circle) corresponding to the time of arising. Dark and light areas on the rim indicate the sleep and wake stages. The average sleep duration (35 records from 7 astronauts) was 6.17±1.38 h. The average amplitude of the circadian rhythm is shown by the length of the vector (‘clock hand’) and the circadian acrophase is indicated by the direction of the vector. The stage of the power-law fractal scaling is least negative during the middle of the rest/sleep span (*ϕ*=−308° and −317°, or 20.5 and 21.1 h after getting up, respectively). The ellipses around the tips of the two vectors are 95% confidence regions for the (*A*, *ϕ*) pairs. As seen from the non-overlap of zero (center of the plot) by the 95% confidence ellipses in Figure 1, the circadian rhythm of *β* is statistically significant: the assumption of no-circadian rhythm (*H*_0_: *A*=0) is rejected on Earth (*P*=0.004) and in space (*P*<0.001). Parameter tests indicate a difference in MESOR, as discussed below, but no difference in the circadian amplitude and acrophase, tested jointly or separately.

### Slope of 1/*f* HRV fluctuations *β*

**Figure 2:**
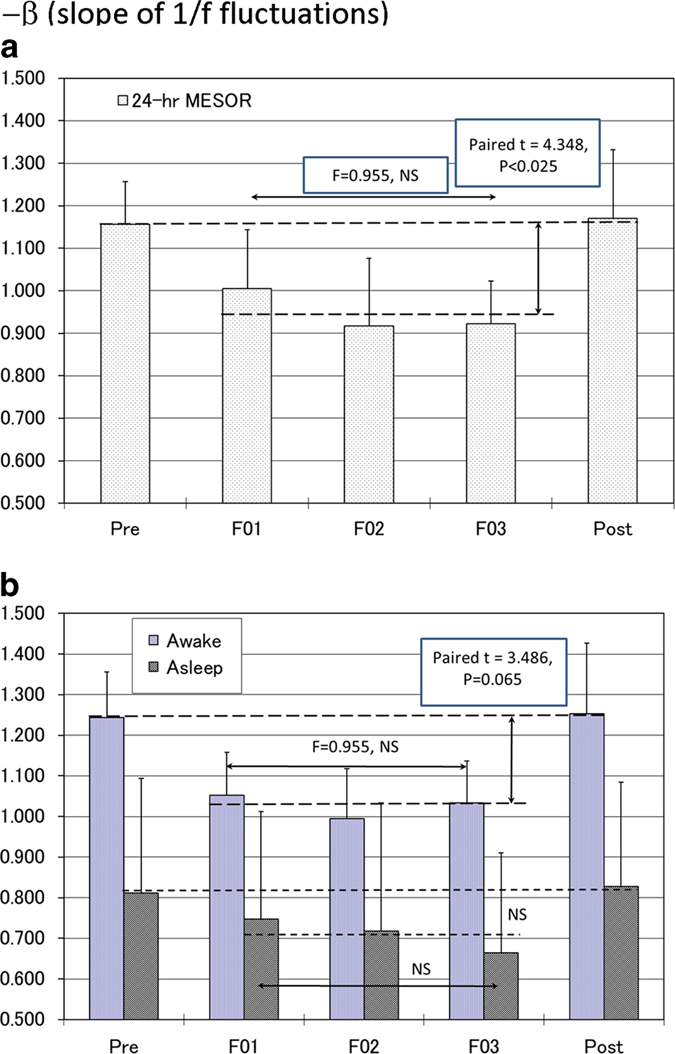
(**a**) Effect of microgravity in space on the 24-h rhythm-adjusted mean (MESOR) of the slope of the power-law scaling, beta. (**b**) Difference in the effect of microgravity on the slope of the power-law scaling, beta, between the awake and asleep spans. It was statistically significant during the awake, but not during the sleep span. Results shown as mean±s.d.

Time series of *β*, the slope of the power-law fractal scaling of NN intervals, analyzed individually by single cosinor to yield estimates of the circadian rhythm parameters, show alterations in space in the microgravity environment. The power spectrum of long-term (analyses over 180-min segments) HRV (1/f^*β*^-type power-law scaling) also exhibits a circadian rhythm in space, but the rhythm-adjusted mean of the slope (*β*) was less negative in space (F01: −1.006±0.137; F02: −0.918±0.159; F03: −0.922±0.101) as compared to on Earth (Pre: −1.156±0.100; Post: −1.170±0.162), Figure 2 (top). The difference is statistically significant (−0.949±0.099 in space vs. −1.163±0.125 on Earth, paired *t*=4.348, *P*<0.025), Table 1. No difference was found among the three records obtained on the ISS (F=0.955, NS).

### Sleep/wake differences in the slope of fractal scaling and in different frequency-domain measures of heart rate variability

Numerically, the slope of the power-law scaling (*β*) was statistically significantly increased during sleep in all records both on Earth and in space, Table 2. After adjustment for multiple testing, the difference was statistically significant on Earth but only reached borderline significance in space, Table 2. The HF component increased during sleep in all records both on Earth and in space, except for one astronaut during F03 when it was slightly decreased. Overall, the difference is statistically significant on Earth but not in space, Table 2. Whereas LF and VLF are numerically increased and ULF is numerically decreased during sleep, differences are not statistically significant, Table 2.

An alteration of the power-law scaling *β* in relation to microgravity in space observed for the MESOR is more pronounced during the awake than during the asleep span, Figure 2 (bottom).

The sleep-wake difference in *β*, Δ*β* (delta beta) is not altered in space: there is no statistically significant difference in Δ*β* among the 5 study stages (mean±s.d., Pre: 0.431±0.282; F01: 0.304±0.297; F02: 0.277±0.280; F03: 0.369±0.282; and Post: 0.426±0.168). The scaling exponent *α* is usually analyzed by detrended fluctuation analysis (DFA), but was here calculated using the equation ‘*α*=(*β*+1)/2’ according to Peng *et al.*^29,30^ Accordingly, Δ*α* (delta alpha) was not altered in space either. The difference of Δ*α* between the awake and asleep spans was larger in our study than previously reported in relation to data collected on the Russian Mir space station.^25^

### Change in different frequency-domain spectral power of heart rate variability during long-term exposure to microgravity

The spectral region most affected by the space environment is the ULF spectral power (ms^2^), which is decreased in space (paired *t*=3.531, *P*=0.060 after adjustment for multiple testing), Table 1. No such change is found for the other 3 components (VLF, LF and HF). The decrease in ULF power is due primarily to changes in ULF band-2 (0.0003–0.001 Hz; 55.5 to 16.6 min; paired *t*=6.967, *P*<0.005) and band-3 (0.001–0.005 Hz; 16.6 to 3.3 min; paired *t*=6.200, *P*<0.01), but not in band-1 (0.0001–0.0003 Hz; 166.7 to 55.5 min; paired *t*=1.325, *P*>0.05), Table 3. ULF band-1 is reportedly^31^ related to the inflammation reaction (CRP) and to the secretion of norepinephrine and cytokine IL-6. ULF band-2 is associated with behavior independent of the intrinsic autonomic regulatory system.^19,20^ ULF band-3 is related in part to the intrinsic autonomic regulatory system (0.001–0.003 Hz) and in part to the VLF component (0.003–0.005 Hz). A lower ULF power in space thus suggests that exposure to microgravity affects the intrinsic autonomic regulatory system of the astronauts in the ISS throughout their long-duration (about 6 months) spaceflight.

## Discussion

The slope of the fractal scaling at frequencies between 0.0001 and 0.01 Hz (periods of 2.8 h to 1.6 min) (*β*) was statistically significantly altered in space. It became less negative (less steep) throughout the 6 months in space as compared with its estimate on Earth (Figure 2a), showing no adjustment during the entire spaceflight. It was restored after return to Earth. This result indicates a major effect of the microgravity environment on the intrinsic autonomic system. Changes observed in the ULF spectral region support this finding. The lack of difference in the other spectral regions (VLF, LF, HF) is in keeping with earlier results from Baevsky *et al*.^32^

As compared with other investigations of ECG records during long-duration spaceflights, our study included repeated 24-h records in space bracketed by similar profiles obtained before launch and after return to Earth. Another study of the autonomic heart rate regulation in male astronauts relying on 24-h ECG records did not include monitoring in space and only compared data over 2-h intervals during the active and rest/sleep spans, focusing primarily on recovery after a mission in space.^33^ Our analysis of whole 24-h records demonstrated that the power-law behavior of HRV remained circadian periodic in space, Figure 1. This result contrasts with heart rate that took 6 months for its circadian rhythm to adapt to the new microgravity environment.^34^

Our results can be interpreted as an alteration of the intrinsic cardiovascular regulatory system related to the microgravity environment of the ISS. Indeed, Aoyagi *et al.*^19,20^ suggested that a power-law behavior *β* of HRV corresponding to periods less than about 1 h reflects an ‘intrinsic’ regulatory system. Hu *et al.*^21,22,35^ confirmed that the scaling exponent *α* of HRV corresponding to periods of about 4 to 60 min exhibits an ‘intrinsic’ circadian periodicity. The change in power-law scaling *β* was more pronounced during wakefulness than during sleep (Figure 2b). Because cardiovascular deconditioning in space can be expected to be most severe during physical exertion when metabolic demands are greatest, it is not surprising that the manifestation of an altered cardiovascular regulating system is primarily seen during wakefulness, as reflected in the results on *β*.

A reduced HRV during long-duration spaceflight was also reported by Xu *et al.*^13^ using linear methods of analysis rather than complexity measures and fractal dynamics, as done herein. Our study found that, on average, *β* was less negative during sleep (between −0.664 and −0.827) than during waking (between −0.994 and −1.253) both on Earth and in space (Table 2). In other words, heartbeat fluctuations in healthy adults were less regular during sleep. This result is in keeping with a previous report in cosmonauts on the Mir space station.^25^ The day–night difference in the scaling exponent *α* calculated^29,30^ from the power-law behavior *β*, however, was larger (from 0.639 to 0.716) than the previously reported^25^ value of ~0.2. Whether the difference can be accounted for by the fact that it was calculated rather than determined by DFA remains to be determined.

Factors underlying the change in *β* during spaceflight may relate to spectral changes observed in the ULF band-2 (0.0003–0.001 Hz) and ULF band-3 (0.001–0.005 Hz), corresponding to periods in the range of 55.5 to 3.3 min (Table 3). Underlying origins and mechanisms, however, remain unclear. In the VLF and ULF frequency bands, including the ULF band-2 and band-3, the power spectrum of HRV is known to follow a 1/*f^β^*-type scaling.^19,36,37^ Both the power in the VLF and ULF components and the slope *β* of fractal scaling are reportedly good predictors of survival not only for patients with coronary artery disease,^37^ but also in healthy subjects.^38^ In view of this potential clinical significance, the origin of the long-period oscillation in HRV has recently been studied.^19,20,39,40^

In order to assess the origin of low frequency fluctuations in HRV in healthy young individuals, Aoyagi *et al.*^20^ minimized environmental and behavioral influences using a constant-routine protocol. These authors reported that in the frequency range below ~10^−3.5^ Hz (periods longer than about 1 h), HRV depends on behavior. The contribution of body movement appears only within a narrow range of frequencies corresponding to periods of ~90 min^19^ reminiscent of Kleitman’s BRAC cycle.^41^ The ULF band-2 and band-3 are not coincident with this frequency range below ~10^−3.5^ Hz, and hence behaviors within body movement probably do not contribute to the alteration of *β* in space. Spectral power in the VLF and ULF range (<0.03 Hz in their case) was reportedly higher during exercise than in resting healthy individuals in 1-h HRV records analyzed by Bernardi *et al*.^39^ Since we found ULF power tending to be lower on the ISS, it is possible that body movements to perform tasks may become slower in space.

Transient changes in the power of the ULF component were reported by Roach *et al.*^40^ to occur around the times bordering sleep in 24-h ambulatory ECG records of healthy subjects. This observation, however, does not account for the consistent decrease in the ULF band-2 and -3 observed in space versus Earth for all seven astronauts. Spectral power decreased by 22.2 to 52.4% in ULF-2 (*P*<0.005) and by 13.2 to 53.9% in ULF-3 (*P*<0.01) during spaceflight as compared to Earth, whereas it increased by 39.4% on average in ULF-1 (*P*>0.05; Table 3). This transposition of variance from ULF-1 to ULF-2 and ULF-3 (and to some extent also in part to VLF), together with the increase in *β* from −1.163±0.075 on Earth to −0.949±0.061 in space (Figure 1 and Figure 2, *P*<0.025) should be taken seriously. Indeed, long-term HRV indices remain relatively stable at various activity levels, making them the most robust measures to assess the cardiac autonomic function during free-running ambulatory conditions. An intrinsic cardiovascular autonomic regulatory system has been robustly maintained in human life.

The cardiovascular system undergoes major changes associated with exposure to microgravity in space, termed a syndrome of cardiovascular deconditioning. The initial trigger to this syndrome is the fluid shift from the lower to the upper body that results in upper body blood volume expansion. This in turn activates Henry–Gauer’s and related reflexes,^18^ leading to a reduction of 2–4% in body mass and of 6–15% in plasma volume in microgravity.^42^ On the other hand, plasma volume contraction has been reported to occur quickly in microgravity, probably as a result of transcapillary fluid filtration into upper-body interstitial spaces. As no natriuresis or diuresis has been observed in microgravity, diuresis cannot account for microgravity-induced hypovolemia.^43^ Moreover, Norsk *et al.*^14^ reported that stroke volume and cardiac output significantly increase by as much as 35 and 41% after 3-month or longer spaceflight. Acclimation of fluid regulation to microgravity thus seems to be more complicated than previously thought.

Structural and functional adaptations occur in the vascular system that could result in impaired responses with demands of physical exertion. Cardiac muscle mass is reduced after flight and contractile function may be altered. Thus, to maintain adequate cardiac function, intrinsic, and reflex cardiovascular responses need to be recruited. It is possible that the change in *β* observed herein indicates that the intrinsic autonomic regulatory system was modified to cope with microgravity conditions in space. An increase in *β* was indeed observed in all three records in space (F01, F2, and F3) in all seven astronauts, and *β* completely recovered after return to Earth (Post), suggesting that exposure to microgravity itself affected and changed the intrinsic autonomic regulatory system.

Subjective self-assessment of sleep quality suggests a slight deterioration in sleep quality during F01 and F02, but not during F03, the worst scores recorded after return to Earth. The effect of microgravity on sleep quality differed among astronauts, with similar scores on Earth and in space for four of them, deterioration for two and improvement for one. Reportedly, sleep quality during spaceflight is not degraded by sleep-disordered breathing.^44^ Decreases in blood pressure and respiratory frequency combined with stable heart rate during prolonged spaceflights reported by Baevsky *et al.*^32^ suggest functional adaptation rather than pathological changes. A recent review^45^ concludes that the lung does not appear to undergo structural adaptive changes when gravity is removed, and hence there is no apparent degradation in lung function upon return to Earth, even after 6 months in space. These observations are in keeping with our finding no difference in HF and LF power on the ISS as compared with Earth in the present study (Table 1).

Recent studies reported, however, that both resting RR-interval and baroreflex responses were well maintained in astronauts during a 6-month spaceflight.^11^ Verheyden *et al.*^10^ also reported that exposure to prolonged microgravity in space induced a shift in the neural mechanism of circulation control toward the ground-based operational point and that this adaptation maintained circulation to a chronically relaxed state for at least 6 months. On the other hand, the microgravity environment in space is associated with cephalic fluid shifts, with increased arterial pressure at the level of the brain and with changes in cerebrovascular structure and function. Long-duration missions on the ISS impaired dynamic cerebrovascular autoregulation.^12^ These observations, together with our findings herein, support the proposition that microgravity is at the origin of an alteration in the intrinsic autonomic regulatory system.

## CONCLUSION

The ‘intrinsic’ cardiovascular regulatory system reflected by the power-law behavior *β* of HRV changed in the ISS throughout the 6-month spaceflight, apparently associated with the microgravity environment. In any serious environmental conditions on Earth, the intrinsic autonomic regulatory system has worked correctly to keep homeostasis and protect life. It is thus critical to improve conditions in space and to ascertain that the regulatory system can function well in space, on the ISS or on another planet as plans are being made to inhabit Mars.

## Materials and methods

### Subjects

Ten healthy astronauts (8 men, 2 women) volunteered for the study. Three male astronauts were excluded from these analyses either because their stay in space was too short or the records did not cover enough of the 24-h span (during the reference stage on Earth and/or upon return to Earth), thus preventing a reliable estimate of the circadian variation, as well as a rigorous individual assessment of changes taking place in association with a microgravity environment. The mean (±s.d.) age of the seven subjects (5 men, 2 women) was 52.0±4.2 years. Their mean stay in space was 172.6±14.6 days. On the average, astronauts had already experienced spaceflight 2.3±0.5 times. The subjects were healthy adults who had passed NASA class III physical examinations. It is unknown whether they were taking any medication since the ethics committee prohibited release of this information. This study obtained consent from all subjects and gained approval from the ethics committee jointly established by the Johnson Space Center and JAXA. A detailed explanation of the study protocol was given to the subjects before they gave written, informed consent, according to the Declaration of Helsinki Principles.

### Experimental protocols

Ambulatory around-the-clock 24-h electrocardiographic (ECG) records were obtained by using a two-channel Holter recorder (FM-180; Fukuda Denshi), which is small (65 [*W*]×18 [*D*]×62 [*H*] mm, 78 g) and useful in the tiny space of a spacecraft. Measurements were made five times: once before flight, three times during flight (F01, F02, and F03), and once after return to Earth. The before-flight measurement session (Pre) was conducted from 469 to 64 days before launch. The three measurement sessions during flight were taken on days 24±5 (F01) and 73±5 (F02) after launch, and 15±5 days before return (F03). The last measurement session was performed 36 to 100 days after return (Post).

Each 24-h ECG record was subdivided into sleep/wake spans determined by checking for altered patterns of NN intervals, as reported previously^46^ and based on the subjects’ 24-h diaries.

### Analysis of heart rate variability

HRV in humans consists of oscillations with periods ranging from seconds to hours.^47^ The measurement procedures and data collection were conducted as previously reported,^34^ briefly summarized as follows. For HRV measurements, QRS waveforms were read from continuous ECG records. The RR intervals between normal QRS waveforms were extracted as the NN intervals. The measured NN intervals were A/D converted with (125-Hz) 8-ms time resolution. The authors first confirmed that all artifacts were actually removed and that the data excluded superventricular or ventricular arrhythmia. Only rare ventricular premature complexes were observed in one male astronaut during the daytime. Frequency-domain measures were obtained with the MemCalc/CHIRAM (Suwa Trust GMS, Tokyo, Japan) software. Time series of NN intervals covering 5-min intervals were processed consecutively, and the spectral power in different frequency regions was computed, namely in the ‘very low frequency (VLF)’, 0.003–0.04 Hz (25 s to 5 min), ‘low frequency (LF)’, 0.04–0.15 Hz (spectral power centered around 10.5 s), and ‘high frequency (HF)’, 0.15–0.40 Hz (spectral power centered around 3.6 s) regions of the MEM spectrum.^34,47,48^ Results representing each HRV component were averaged over the entire 24-h (about 288 values), asleep (about 72 values) and awake (about 216 values) spans. The VLF component is considered to represent endocrine activities, particularly the renin–angiotensin system, which regulates vasomotor activities.^39,49^

### Measurement of 1/*f* fluctuations in HR dynamics

**Figure 3:**
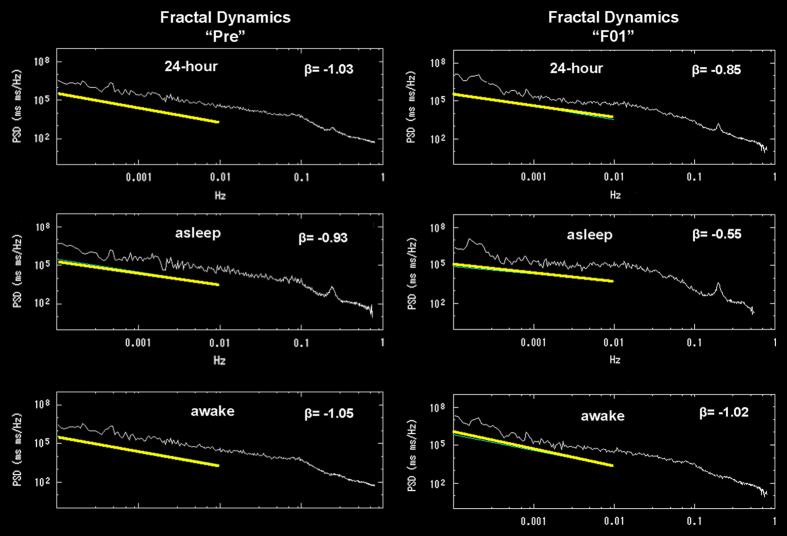
Power spectra of long-term heart rate variability focusing on the frequency range of 0.0001–0.01 Hz (periods of 2.8 h to 1.6 min). Left: cardiovascular fractal dynamics assessed by the slope of fractal scaling *β* (beta) before flight (Pre); top: 24-h span; middle: sleep span; bottom: awake span. Right: cardiovascular fractal dynamics assessed by *β* (beta) during F01 (days 24±5 of flight); top: 24-h span; middle: sleep span; bottom: awake span.

Time series of NN intervals were processed consecutively in 180-min intervals, progressively displaced by 5 min, to estimate the ‘ultralow frequency’ (ULF) component (0.0001–0.003 Hz; periods of 2.8 h to 5 min), including the following three components: ‘ULF band-1’, 0.0001–0.0003 Hz (166.7 to 55.5 min), ‘ULF band-2’, 0.0003–0.001 Hz (55.5 to 16.6 min), and ‘ULF band-3’, 0.001–0.005 Hz (16.6 to 3.3 min). To evaluate the 1/*f^β^*-type scaling in HRV, the log_10_(power) (ordinate) was plotted against log_10_(frequency) (abscissa) and a regression line fitted to estimate the slope *β*, as reported earlier.^50^ Focus was placed on the frequency range of 0.0001–0.01 Hz (periods of 2.8 h to 1.6 min; Figure 3). Results were averaged over the entire 24-h (about 250 estimates), asleep (about 70 estimates) and awake (about 180 estimates) spans.

The ULF component mainly includes non-periodic (nonlinear) components that are not easily connected to background factors. Some investigators^31^ have suggested that it is related to the inflammation reaction (CRP) or to the secretion of cytokines (IL-6).

### Cosinor analyses

Data series collected on Earth and in space were fitted with a 24-h cosine curve,^51^ yielding estimates of the MESOR (Midline-Estimating Statistic of Rhythm, i.e., a rhythm-adjusted mean), amplitude (half the predictable extent of change within 24 h) and acrophase (a measure of the timing of overall high values within 24 h). Individual rhythm characteristics were summarized by population-mean cosinor and compared by parameter tests.^51–53^ Code is available at corne001@umn.edu.

### Statistical analyses

This study attempts to answer three specific questions: (a) are HRV indices, including *β*, different in space than on Earth; (b) are HRV end points changing (adjusting) while in space; and (c) is there a difference in HRV end points between the awake and asleep spans.

Data were expressed as mean±s.d. The two-sided paired *t*-test was used for the space versus Earth and for the asleep versus awake comparisons. In order to further minimize inter-individual variability, log ratios of individual averages in space versus Earth were used for comparison. A one-way ANOVA for repeated measures was used to compare HRV end points among the three recordings obtained on the ISS. Analyses were carried out using Stat Flex (Ver. 6) software (Artec, Osaka, Japan). A *P*-value <0.05 was considered statistically significant. *P*-values were adjusted for multiple testing using Bonferroni’s inequality.

In all tests, homogeneity of variance was satisfied by the F_max_-test and by Bartlett’s test in all but one case that deviated from homogeneity only very slightly.

## Figures and Tables

**Table 1:** Changes in the slope (*β*) of power-law fractal scaling and of frequency-domain measures of heart rate variability during 6-month mission in space (from 24-h ECG records)

	*Case 1*	*Case 2*	*Case 3*	*Case 4*	*Case 5*	*Case 6*	*Case 7*	*Mean*	*s.d.*	*Space versus Earth: paired* t *(*P*)*^a^	*F01–F03 one-way ANOVA F (*P*)*
Age (years)								52.0	4.2		
Days on ISS								172.6	14.8		
*N* previous spaceflights								2.3	0.5		
Wt (kg)								76.0	13.6		
Ht (m)								1.75	0.07		
BMI (kg/m^2^)								24.5	2.8		
											
*β*											
Pre	−1.078	−1.155	−1.287	−1.183	−1.278	−1.030	−1.082	−1.156	0.100	4.348 (<0.025)	0.403 (NS)
F01	−0.899	−1.093	−1.206	−0.967	−0.904	−1.130	−0.842	−1.006	0.137		
F02	−0.690	−0.835	−1.039	−1.097	−0.829	−0.838	−1.096	−0.918	0.159		
F03	−0.798	−0.964	−1.099	−0.889	−0.880	−0.845	−0.980	−0.922	0.101		
Post	−0.985	−1.101	−1.245	−1.161	−1.490	−1.077	−1.132	−1.170	0.162		
											
*ULF*											
Pre	1394.5	3617.1	3152.5	3219.9	2520.2	6363.2	9936.1	4314.8	2903.5	3.531 (0.060)	0.472 (NS)
F01	1392.1	2575.8	3650.0	3949.0	1065.5	6975.8	3940.7	3364.1	1983.0		
F02	795.3	1199.0	2140.9	2328.3	1534.0	2824.9	6054.5	2411.0	1750.0		
F03	2140.0	2473.1	2743.5	1801.6	1626.8	3697.7	7242.4	3103.6	1950.0		
Post	1643.7	3545.4	3811.4	3001.1	3542.7	4898.3	7922.4	4052.1	1965.3		
											
*VLF*											
Pre	937.1	1421.7	1257.0	1635.0	715.0	4054.7	5276.2	2185.2	1756.7	2.066 (NS)	0.210 (NS)
F01	1174.8	965.7	1118.1	2204.9	761.2	2494.8	3312.4	1718.8	959.1		
F02	1242.1	1176.0	1103.3	1103.2	753.0	2978.1	2376.0	1533.1	815.4		
F03	1158.4	1576.0	1247.4	1385.4	760.8	3177.0	3810.3	1873.6	1148.8		
Post	1372.8	2089.7	1618.8	1569.4	652.5	2920.2	3936.7	2022.9	1091.0		
											
*LF*											
Pre	573.80	537.50	653.00	753.50	157.86	1341.30	866.10	697.58	360.59	0.761 (NS)	0.051 (NS)
F01	374.15	537.80	535.10	1190.60	135.99	1052.90	745.90	653.21	371.89		
F02	360.89	646.60	482.12	851.40	203.03	1173.20	570.90	612.59	322.06		
F03	323.51	823.50	571.80	980.10	177.68	1142.60	672.30	670.21	345.24		
Post	498.64	848.10	636.26	758.80	137.86	1000.60	800.00	668.61	282.68		
											
*HF*											
Pre	107.16	113.62	52.18	163.19	37.62	192.85	164.67	118.76	58.81	0.586 (NS)	0.435 (NS)
F01	85.90	90.92	55.91	233.26	21.79	147.03	157.70	113.22	71.20		
F02	70.90	98.68	73.52	157.51	31.22	178.46	100.44	101.53	51.20		
F03	171.49	127.72	69.54	220.87	30.29	192.50	119.11	133.07	67.75		
Post	92.04	192.28	99.84	154.62	23.64	114.60	137.34	116.34	53.44		

Abbreviations: ANOVA, analysis of variance; BMI, body mass index; F, female; HF, high frequency; Ht, height; ISS, International Space Station; LF, low frequency; M, male; NS, not signigicant; Pre, before-flight measurement session; Post, after-return measurement session; ULF, ultralow frequency; VLF, very low frequency; Wt, weight; y, years.

a Comparison of log(Space/Earth) ratios.

**Table 2:** Day–night differences in the slope (*β*) of fractal scaling and in various frequency-domain measures of heart rate variability

	*Study stage*	n	*24-h MESOR*		*Awake*		*Asleep*		*Difference between awake and asleep*	
			*Mean*	*s.d.*	*Mean*	*s.d.*	*Mean*	*s.d.*	*Paired* t	P*-value*
Slope of 1/*f*	Pre	7	−1.156	0.100	−1.243	0.113	−0.811	0.282		
	F01	7	−1.006	0.137	−1.051	0.106	−0.747	0.265		
	F02	7	−0.918	0.159	−0.994	0.124	−0.717	0.316		
	F03	7	−0.922	0.101	−1.033	0.104	−0.664	0.247		
	Post	7	−1.170	0.162	−1.253	0.174	−0.827	0.258		
	Earth	7	−1.163	0.125	−1.248	0.140	−0.819	0.255	5.409	<0.010
	Space	7	−0.949	0.099	−1.026	0.077	−0.710	0.252	3.261	0.085
ULF component (0.0001–0.003 Hz)	Pre	7	4314.8	2903.5	5364.4	4808.4	2737.8	1434.0		
	F01	7	3364.1	1983.0	3641.4	1613.1	2334.0	1404.0		
	F02	7	2411.0	1750.0	3295.4	3126.0	1954.5	1480.2		
	F03	7	3103.6	1950.0	4620.6	3590.3	2112.9	1068.8		
	Post	7	4052.1	1965.3	4415.2	2108.0	2818.3	1328.7		
	Earth	7	4183.5	2416.9	4889.8	3410.0	2778.1	1373.7	2.351	NS
	Space	7	2959.6	1617.3	3852.5	2517.4	2133.8	1187.9	1.853	NS
VLF component (0.003–0.04 Hz)	Pre	7	2185.2	1756.7	2184.4	1857.4	3501.6	3699.9		
	F01	7	1718.8	959.1	1847.6	972.6	2699.4	2027.5		
	F02	7	1533.1	815.4	1592.7	718.7	2167.6	1738.8		
	F03	7	1873.6	1148.8	1878.6	1102.5	3211.2	3326.0		
	Post	7	2022.9	1091.0	2011.8	1113.9	3226.8	2977.3		
	Earth	7	2104.1	1408.9	2098.1	1471.3	3364.2	3330.0	1.201	NS
	Space	7	1708.5	932.8	1773.0	893.9	2692.7	2267.4	1.277	NS
LF component (0.04–0.15 Hz)	Pre	7	697.6	360.6	680.5	321.7	884.0	609.6		
	F01	7	653.2	371.9	638.0	352.9	832.2	614.0		
	F02	7	612.6	322.1	563.5	262.0	806.5	545.5		
	F03	7	670.2	345.2	600.7	288.7	956.3	638.8		
	Post	7	668.6	282.7	632.5	247.8	985.5	686.6		
	Earth	7	683.1	309.7	656.5	270.4	934.8	647.0	1.577	NS
	Space	7	645.3	337.3	600.7	293.6	865.0	595.3	1.762	NS
HF component (0.15–0.40 Hz)	Pre	7	118.8	58.8	102.7	49.5	188.5	115.2		
	F01	7	113.2	71.2	100.7	64.8	150.0	89.2		
	F02	7	101.5	51.2	87.1	40.4	148.0	93.6		
	F03	7	133.1	67.8	106.9	48.0	222.7	162.2		
	Post	7	116.3	53.4	104.6	51.0	181.5	73.8		
	Earth	7	117.5	50.0	103.7	43.9	185.0	89.3	3.737	0.050
	Space	7	115.9	59.8	98.3	49.2	173.6	107.4	2.944	0.130

Abbreviations: HF, high frequency; LF, low frequency; MESOR, Midline-Estimating Statistic of Rhythm; NS, not significant; ULF, ultralow frequency; VLF, very low frequency; Pre, before-flight measurement session; Post, after-return measurement session.

**Table 3:** Changes in frequency-domain measures of heart rate variability during 6-month mission in space

		*Pre*	*F01*	*F02*	*F03*	*Post*	*Space versus Earth: Paired* t *(*P*)*	*F01*–*F03: F (*P*)*
*24-h*								
ULF component (0.0001–0.003 Hz)	Mean s.d.	4314.8 2903.5	3364.1 1983.0	2411.0 1750.0	3103.6 1950.0	4052.1 1965.3	3.531 (0.060)	0.472 (NS)
ULF band-1 (0.0001–0.0003 Hz)	Mean s.d.	1669.2 1138.7	1822.4 880.8	1494.2 1302.6	2257.5 1566.4	1391.5 694.7	1.325 (NS)	0.625 (NS)
ULF band-2 (0.0003–0.001 Hz)	Mean s.d.	1708.6 1578.9	892.5 420.1	897.6 637.7	939.7 606.3	1443.0 644.1	6.967 (<0.005)	0.015 (NS)
ULF band-3 (0.001–0.005 Hz)	Mean s.d.	1748.3 1582.1	961.3 588.5	875.8 532.4	1087.5 896.3	1596.6 1009.0	6.200 (<0.01)	0.166 (NS)

Abbreviations: NS, not statistically significant; ULF, ultralow frquency.
